# Evidence for a Priori Existence of Attentional Bias Subgroups in Emotional Processing of Aversive Stimuli

**DOI:** 10.3389/fnbeh.2017.00087

**Published:** 2017-05-12

**Authors:** Casper H. van Heck, Joukje M. Oosterman, Kim M. A. de Kleijn, Marijtje L. A. Jongsma, Clementina M. van Rijn

**Affiliations:** ^1^Donders Institute for Brain, Cognition, and Behaviour, Radboud UniversityNijmegen, Netherlands; ^2^Faculty of Social Sciences/Behavioral Science Institute, Radboud UniversityNijmegen, Netherlands

**Keywords:** attention, EEG, attentional bias, dot probe, behavior, avoidance, hypervigilance

## Abstract

Little is known regarding inter-individual differences in attentional biases for pain-related information; more knowledge is crucial, since these biases have been associated with differences in pain processing as well as in predicting the risk of postoperative pain. The present study investigated EEG correlates of attentional bias patterns for pain-related information, with specific focus on avoidance- and vigilance-like behavior. Forty-one participants performed a dot-probe task, where neutral and pain-related words were used to create neutral, congruent, incongruent, and double (two pain-related words) trials. EEG was recorded, which was used to generate ERP's of the word-processing phase and the post-dot phase. Participants were placed in two subgroups based on the direction of their attentional bias (either positive; toward the pain-related words, or negative; away from pain-related words). Using t-profiles, four latency windows were identified on which the two subgroups differed significantly. These latency windows yield areas which correspond with the P1-N1 domain and the P3b for the word-processing phase, while the post-dot phase latency windows cover the areas of the P200 and the P3b. The two subgroups show differences on congruent, incongruent, and the double trials, but interestingly also on the neutral trials. Most notably, the area in the word-phase associated with the P3b is diminished in the subgroup showing a negative bias. The deflections associated with both early and late attentional components, including the P3B, as well as a positive deflection in the timeframe of proposed response evaluation processes differ significantly between subgroups. In this study we demonstrated that different attentional biases exist in the healthy population, by showing differences in ERP's. We also show differences in processing neutral trials, which suggests there are fundamental differences between these groups in processing words in general.

## Introduction

Nociceptive stimuli are amongst the most prominent and reliable aversive stimuli. As these stimuli alert us to an actual or potential (perceived) immediate threat, they are therefore capable of rigorously directing and manipulating attention (Keogh et al., 2001b; Keogh and Cochrane, 2002; Dittmar et al., 2011).

However, individuals have been observed to have attentional biases toward or away from pain and pain-related information. These biases are commonly grouped under avoidance or hypervigilance, based on the direction of the bias. A bias away from non-neutral information can be termed “avoidance,” while a bias toward non-neutral information can be termed “hypervigilance.”

It has been demonstrated that these attentional biases can affect pain sensitivity and augment pain-related behaviors, and both avoidance and hypervigilance have been linked to the processing of pain-related information (Koyama et al., 2005; Hakamata et al., 2010; Schoth et al., 2012; Herbert et al., 2013).

Moreover, these two different attentional biases have clinically relevant implications. For example, hypervigilance has been associated with a high sensitivity to nociceptive stimuli (Geringer and Stern, 1986), leading to higher clinical pain severity (Wilner et al., 2014). In addition, avoidance has been shown to increase the chances of developing chronic pain (Vlaeyen and Linton, 2000), as well as affecting the recovery process (Vlaeyen and Crombez, 1999). Moreover, individual differences in both avoidance-like behavior as well as hypervigilant behavior have been shown to be valid predictors of postoperative pain (Goodin et al., 2009; Lautenbacher et al., 2009, 2010, 2011; Pulvers and Hood, 2013; Grosen et al., 2014; Wong et al., 2014).

There is also evidence of multiple coexisting attentional biases in the healthy population. For example, it has been demonstrated that a propensity toward avoidance predicted a lower risk of future post-traumatic stress in a population of healthy combat soldiers during training (Lin et al., 2015).

Most documented individual variations are gathered using paradigms based on reaction time (RT) differences. While these are commonly used and a generally accepted method of studying attentional effects (MacLeod et al., 1986; Keogh et al., 2001a; Baum et al., 2011), its use is limited; it cannot show if the attentional bias reflects a transient response tendency to a stimulus or a more general personality trait.

To investigate if participants with different attentional biases differ only in response tendency or show also more general differences in the processing of emotional stimuli, neuroimaging methods might be used. For this, EEG is ideal, as it is an established and proper neuroimaging method, as well as easy to implement in existing experiments. Moreover, by extracting the Event-Related Potential (ERP) from the ongoing EEG time locked to stimulus presentation, inferences about differences in stimulus processing can be made. Furthermore, the distinctive peaks and troughs of these ERPs have been extensively linked to different cognitive processes arising from different functional neural circuits, which has resulted in a wealth of research concerning the relevance and functionality of specific deflections (Treede et al., 1999; Luck, 2005; Nikendei et al., 2005).

Most studies of the attentional systems which include EEG focus on a subset of ERP components, mainly the mid-latency ERP components that occur between 50 and 150 ms after stimulus presentation, such as the P1 and N1. Though predominantly determined by the stimulus characteristics, these components are also sensitive toward top-down modulations, such as changes in attention, especially when exposed to stimuli with an emotional content (Lee et al., 2009; Van der Lubbe et al., 2012). These components also seem to be dissimilar between individuals; high-anxious individuals have been shown to have increased amplitudes of these deflections, while their latencies tend to be decreased.

One of these components, the N1, has been suggested to reflect a sensory gain control mechanism (Luck et al., 2000), and can be observed to be increased in amplitude based on the level of threat or emotional content (Santesso et al., 2009; Brosch et al., 2011).

The P1, which normally precedes the larger N1, has been shown to have a similar relationship with emotional content as the N1, but it has also been suggested that this component can be influenced by individual characteristics, such as vigilance (Dittmar et al., 2015). The P1-N1 complex is thought to originate from parietal-temporal-occipital regions, although some results suggest that it may also be generated by frontal regions (Clark et al., 1994).

A later set of components also shows to be affected by emotional content; the N2 component as well as a late positive component were significantly increased based on the “threat” of the trial (Kappenman et al., 2013). This specific N2 component (as well as the earlier N1) has been used as an indicator of attentional selectivity before (Eimer, 1996). However, the N2 has also been shown to be increased in anxious vs. non-anxious individuals, regardless of emotional content in the provided stimuli (Eldar et al., 2010). The N2 is thought to be generated frontally, and may reflect frontal control of the visual system (Luck and Hillyard, 1994).

The late P3 component has been shown to be increased in response to anger-related stimuli, which the authors link to the P3's relationship with target evaluation and response selection (Eldar and Bar-Haim, 2010). The P3 has been linked to late-stage higher-order functions, but recent studies have stated that this component can be separated into the P3a and the P3b, where the first is associated with stimulus-driven attentional mechanisms, while the P3b is more related to event categorization, attention, memory processing, and target evaluation (Kok, 2001; Polich, 2003, 2007). Moreover, the P3b has been shown to be reduced in amplitude for unpleasant visual stimuli, together with confirming the links between emotional content and the P1 and P2 components (Delplanque et al., 2004). The source of the P3b is unclear, but it is commonly found near the parietal areas, and together with the preceding P3a is implied in an attention and/or memory-related circuit pathway between the frontal and the temporal/parietal areas (Polich, 2003).

Studies that have applied EEG to illustrate inter-individual differences concerning attentional biases toward pain-related stimuli are scarce, and yield only few significant results (Dittmar et al., 2011). As a result, there is a lack of knowledge regarding the presence of these biases in the healthy population, while there are indications that inter-individual differences of attentional biases are clinically relevant.

In this report, we explore ERP's of participants performing a dot-probe task using pain-related stimuli. After separating the population based on the direction of their bias (either toward, or away from pain-related information), we will explore possible differences in ERP's.

We employed a dot-probe paradigm in which word pairs were presented, followed by a dot, to which the participant is to respond. The dot appeared at the same location of one of the two words within the word pair, and a delay (or speeding up) in responding to the dot is usually observed due to the direction of the attentional bias combined with the meaning or content of the word. For example, in individuals prone to hypervigilance, a pain-related word might capture attention long enough to show a markedly faster response if a dot is presented at the same location. In contrast; an individual showing primarily avoidance might direct attention away from a threatening word, which would result in a slower response if the dot appears at the location of that word, but a faster response if the dot is presented at the location of the opposite word.

- The primary goal of the present study was to examine whether differences in attentional bias, based on response latencies to the dot, are already present in the early word-processing phase. Differences in this phase would suggest that there is an à priori difference between the two subgroups.
- The secondary goal is to investigate the post-dot phase for similar differences, but then based on the differences between congruent and incongruent trials. Differences in this phase due to increased vigilance or avoidance are expected to be reflected in the ERP components as well.

## Materials and methods

### Participants

Participants were recruited from a population of healthy students of the Radboud University Nijmegen, who were required to gather academic credits through participation in studies. Students not eligible for these points received monetary compensation. The study included 41 participants (16 male, 25 female), aged 21 (*M* = 21.20, SD = 2.67, Range = 17–29).

Participants were subject to exclusion criteria, such as diabetes, cardiovascular problems, depression, chronic pain (now and in the past), addiction (now and in the past), and pain at the moment of or during the days leading up to the experiment. Participants were also excluded if they were receiving treatment from a medical specialist or were seeing a psychologist, or if they were using psychoactive medication for any reason.

This study was approved by the Ethic Committee Social Sciences (registered under ECG2012-1301-005) of the Radboud University Nijmegen, and was performed in accordance with the requirements of the Declaration of Helsinki. All subjects signed a standard written informed consent.

### Setup

The dot-probe experiment, as used here, was based on the version described by Keogh (Keogh et al., 2001b; also see the “Stimuli”-Section). The software Presentation® from Neurobehavioral Systems (Version 18.3, www.neurobs.com) was used to run the experiment. RTs were measured using a Logitech G510S Gaming Keyboard, which has a response time < 2 ms and an accuracy of 1 ms.

### EEG

We used a standard EEG setup, which consists of a BrainProducts ActiCap 32-channel EEG system. Electro-oculogram (EOG) was recorded using a BrainProducts ExG extension, which ensured the EOG-channels were not included in the common average reference (i.e., the EOG-channels were not electrically linked to the other channels).

All signals were recorded with a sample frequency of 5,000 Hz, with the impedance below 20 KΩ for all channels. The BrainAmp amplifier has two built-in electronic filters; one high-pass filter of 0.016 Hz, and one low-pass filter of 1,000 Hz. Montage of the electrodes was according the 10–20 system (Oostenveld and Praamstra, 2001).

Due to the dot-probe paradigm consisting of multiple stimuli per trial, the resulting ERP's will be compound ERP's. Therefore, our approach will be partially data-driven, meaning intervals of interest will be localized solely based on their statistical properties. Finally, these regions of interest will be linked to existing research.

### Stimuli

We gathered 60 pain-related words from the McGill pain questionnaire and from previous studies. The pain-related words were all adjectives, with their lengths conforming to a normal curve (*M* = 8.7, σ = 1.5). Comparing their valence and arousal ratings with the ratings of the neutral words, by using the database generated by Moors et al. (2012), showed their ratings to be higher than those of the neutral words, with a mean valence of 3.9 (σ = 0.8) vs. 2.3 (σ = 1.1), and a mean arousal of 4.5 (σ = 1.1) vs. 0.7 (σ = 0.3).

The neutral words were sourced from the subtitle database maintained by the Center for Reading Research of Ghent University (Keuleers et al., 2010). Only adjectives without additional meanings or alternative interpretations were selected, which were also matched in length and usage frequency to the pain-related words. The resulting list was passed to three native Dutch speakers for additional verification. A total of 209 words remained for use as neutral stimuli (see Supplementary Material).

**Figure 1:**
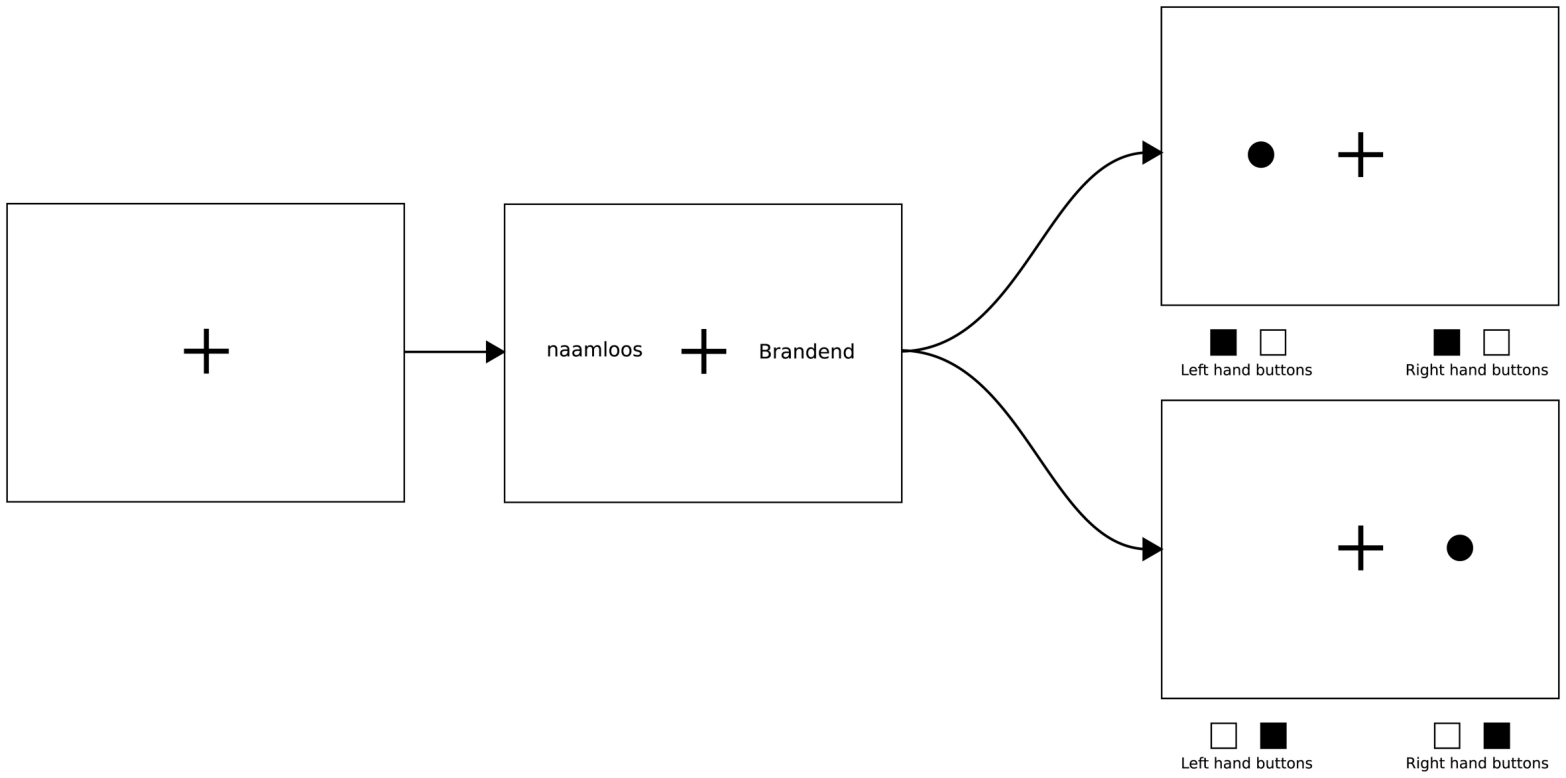
**An example of a full trial**. The first (leftmost) image shows an “empty” screen, with the fixation cross only. The second (center) image shows a typical non-neutral trial, using a neutral word (“naamloos,” meaning not having a name) and a pain-related word (“brandend,” meaning “burning” or “a burning sensation”). The two remaining (rightmost) images show two possible outcomes; the top option shows the dot appearing on the left side, which would make this trial an incongruent trial, while the bottom option shows the dot appearing on the right side, which would make this a congruent trial. Note the desired response shown below the two rightmost images; the participant is to respond to the location of the dot with both hands (the correct response is shown as filled squares).

As is common in a dot-probe experiment, blocks consisted of congruent, incongruent, and neutral trials. These trials are constructed using two words, one on each side of a monitor (with a fixation cross in the middle of the monitor, see Figure 1). The words are shown for a specific amount of time, and then disappear, after which the dot appears at the location of either the left or the right word. Congruent and incongruent trials consist of one neutral word and one non-neutral (in this study: pain-related) word. If the subsequent dot appears on the position of the pain-related word, the trial is “congruent,” and if the dot appears on the position of the neutral word, the trial is “incongruent.” To establish a proper baseline condition, neutral trials need to be included, which are made up of two neutral words.

Also included in the experiment were double trials, using two pain-related words. The reason for including this type of trial was that one could propose that the type of attentional bias influences how such trials are processed. For example, these trials may be perceived as stressful for participants showing avoidance-like behavior, as they are unable to avoid the pain-related stimulus. Consequently, differences in brain ERPs between the two attentional bias groups during presentation of the words can be expected.

### Procedure

To ensure cognitive processing of the words took place, we explicitly told the participants there would be a questionnaire concerning the words at the end. In doing so, we attempted to ensure that the participants paid attention to the words.

The experiment was divided into four blocks, and contained three breaks of 5 min. The impedance was checked during every break.

The trials consisted of the following three parts:

1. A baseline period with just a fixation cross. This period lasted for a minimum of 1,500 ms, and a maximum of 2,000 ms. Note that the fixation cross was continually present.
2. A word-phase, during which the words were displayed. As can be seen in Figure 1, one word was presented on the left, and one on the right side of the fixation cross. These words were horizontally aligned and placed with their centers on a fixed distance from the center of the fixation cross. This phase lasted exactly 500 ms.
3. The “post-dot”-phase, where the words were replaced by a single dot, which appeared at the location of one of the two words. In this phase, participants were required to indicate where the dot had appeared as quickly and accurately as possible by pressing two of four possible response buttons, using both hands (also see Figure 1). Both hands were used to eliminate lateralized motor activity, to make it easier to detect other lateralized activity.

The RTs of both hands were recorded for each trial, and were averaged into a single value for analysis. Trials with large (>50 ms) differences in RTs between the left and right hand were removed.

During visual presentation, the pain-related words were paired with neutral words, creating non-neutral trials, which can be separated into either congruent (dot on the non-neutral word) or incongruent (dot on the neutral word) trials. The number of neutral trials between the non-neutral trials varied randomly between 1 and 4. Each participant was exposed to a total of 60 non-neutral trials, using all 60 pain-related words once, of which 30 on the left and 30 on the right side of the fixation cross.

Neutral words were randomly selected for each trial, and were not allowed to repeat within 10 consecutive trials. Trials were generated in a list-based format beforehand, using Matlab®, and checked manually before use.

It should be noted that participants were not informed about the precise study goals, and all mention of pain-related outcomes (such as through pain-related questionnaires) was saved for the end of the experiment. This was done to ensure the participant was not aware of the reasoning behind the experiment beforehand.

### Subgroup split

Two subgroups were created, through the established method of the bias index (Asmundson et al., 2005a,b; Roelofs et al., 2005; Sharpe et al., 2009; Haggman et al., 2010). This index relies on comparing the responses to the congruent and incongruent trials, and can be calculated by using the following formula:

$$\begin{array}{l} \frac{\left(\text{RT}(\text{tl},\text{ dr})\;-\;\;\text{RT}(\text{tr},\text{ dr}))\;+\;(\text{RT}(\text{tr},\text{ dl})-\;\text{RT}(\text{tl},\text{ dl})\right)}{2} \end{array}$$

Here, RT stands for the mean of the reaction time for a specific stimulus type. The different stimulus types are defined by the letters between the brackets; “t” stands for target, “d” for dot, and “l” (left), and “r” (right) represent the location on the screen. This method is commonly used in studies on attentional biases (Asmundson et al., 2005a,b; Roelofs et al., 2005; Sharpe et al., 2009; Haggman et al., 2010). Using this, participants can be placed in two possible subgroups:

- Participants with a **positive bias**, who respond **faster** on the **congruent trials** than on the **incongruent trials**. These participants primarily display **vigilance**-like behavior.
- Participants with a **negative bias**, who respond **faster** on the **incongruent trials** than on the **congruent trials**. These participants primarily display **avoidance**-like behavior.

However, not all participants are expected to show a clear bias; some participants might not have an attentional bias, or might simply not read the words. All participants showing a bias of 10 or less milliseconds were termed the “no bias”-subgroup (*n* = 9), and were removed from the analysis.

### EEG analysis

To detect baseline-differences between groups, which may influence our results, 3 min of resting EEG was recorded. The frequency properties of the resting EEG of the two groups was compared.

The EEG was analyzed using Matlab®, with the Fieldtrip analysis package (Oostenveld et al., 2011). A Fourier transform of the resting EEG was used to investigate possible resting state differences between the two groups.

After initial pre-processing, using a 1 Hz high-pass filter, and a 40 Hz low-pass filter, the EEG was segmented according to trial onset, and baseline correction was applied. Trials were visually inspected for artifacts, and trials found flawed or significantly polluted were removed. Please note that in the whole sequence of events, different time regions were investigated. In order to investigate these different regions, each event was time-locked to different moments. As a result, the baseline correction was repeated several times, each time with a different baseline time-epoch.

For the word-interval, the baseline was defined as −250 to 0 (leading to a total baseline of 250 ms), and the investigated interval ranged from 0 to 500 ms, during which the words were present. During the presentation of the words, the congruency of the trial is not a factor, since the dot has not appeared yet, and therefore the congruent and incongruent trials were combined into “single” trials. As a result, in this interval, there are three conditions; neutral (no pain-related words), single (a single pain-related word), and double (two pain-related words).

Since the dot-appears at *t* = 500 ms, the investigated interval post-dot was defined as ranging from 500 to 1,500 ms. The baseline was set at −500 to + 500, which equates to a full second of baseline over the 500 ms word-period as well as 500 ms in the period with the fixation cross. This was done to reset the baseline, since the delivery of the fixation cross and the words introduces their own EEG perturbations. The appearance of the dot introduces new information to the subject, which separates the single trials into congruent and incongruent trials. As a result, in this interval, there are four conditions; neutral, congruent, incongruent, and double.

For all 41 subjects, for each condition, an averaged ERP was produced. To enable pair-wise comparison of the conditions we used t-profiles (Krijzer and van der Molen, 1987), which are *t*-tests between the sets of individual averages on every time point. We averaged the t-profiles of all conditions into a single Grand Average (GA) t-profile. In this GA t-profile, intervals during which the t-reached significance were identified, which were then used to create latency windows. Only clusters with a minimum of 20 subsequent significant time points were considered as a potential latency window, in order to avoid type II errors.

In the figures, Grand Average ERPs are shown, which are created by collecting the individual average ERPs into a single average. The t-profiles are shown together with the Grand Averages ERPs of both groups.

### EEG statistics

Since ERP differences appeared to be maximal over the Pz electrode, which is not unexpected since some of the relevant deflections have been known to originate in the parietal area (Bledowski et al., 2004; Polich, 2007), only the data from Pz were further analyzed.

Statistical analysis was performed on values extracted from these average ERPs, using the latency windows provided by the t-profiles. This led to every participant having a single value per condition per latency window.

Statistical analysis of these latency window-based values was performed using repeated-measures GLM's, where the conditions are treated as within-subjects variable and group as between-subjects variable. For the word-phase a 2 (attentional bias subgroup: positive, negative) × 3 (condition: neutral, single, double) GLM was run. For the dot-phase a 2 (attentional bias subgroup: positive, negative) × 4 (condition: neutral, congruent, incongruent, double) GLM was performed. Because the main outcome of the dot-probe paradigm relates to the difference between congruent and incongruent trials, a special contrast will be added which compares the two, as well as contrasts that compare the different conditions with the neutral “baseline” condition.

### Number of trials

After the exclusion of trials containing artifacts, on average 28.9 (SD = 3.90) trials could be used for the congruent condition, and on average 29.2 (SD = 3.54) trials for the incongruent condition. It has been shown that an average consisting of 20 trials is of sufficient quality to base conclusions on Cong et al. (2011). As such, we are confident there are no issues with the trial counts in making up the averages.

### Subgroup properties

**Figure 2:**
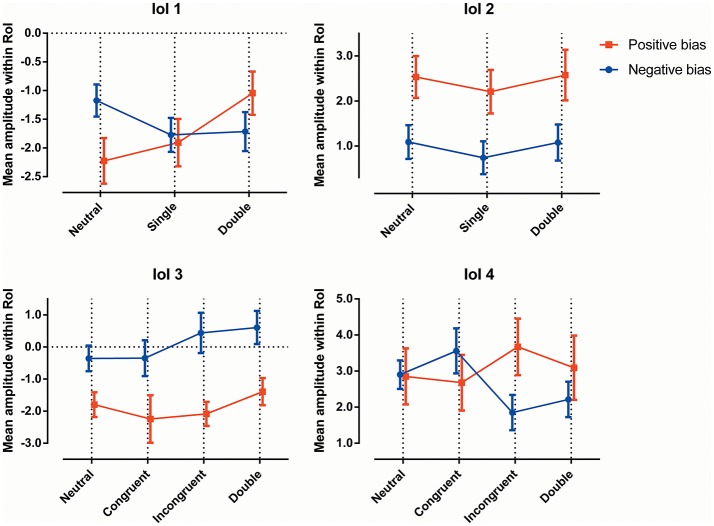
**The ERP amplitudes within each latency window, for all conditions, for both attentional bias directions separately**.

Individuals were split into subgroups as explained earlier, with participants showing no substantial bias excluded from both subgroups. The resulting subgroups consisted of 19 individuals with a negative attentional bias, and 13 individuals with a positive bias. See Figure 2 for a visualization of the amplitudes in the latency windows, and see Table 2 for the average reaction times of the groups for all conditions.

## Results

### Resting EEG

Statistical analysis of the Fourier transforms revealed no significant difference in the resting EEG between the subgroups.

### Word-phase ERPs

#### First (early) latency window: 84–92 ms

Eighty-eight milliseconds after the appearance of the words, the t-profile indicates the presence of a latency window.

A repeated-measures GLM was conducted on the data with “condition” as within-subjects and “subgroup” as between-subjects factor. As can be seen in Table 1, this analysis showed no main effect of “subgroup” or “condition,” but a significant interaction of the two. *Post-hoc* contrasts suggest this difference between the groups to be mainly present on the neutral and the double trials.

**Table 1:** **Reaction time data for the two subgroups, as well as the “uncommitted” or “no bias” population**.

	**Neutral**		**Congruent**		**Incongruent**		**Double**	
	**Mean**	**Std**	**Mean**	**Std**	**Mean**	**Std**	**Mean**	**Std**
No bias	460.74	38.81	456.81	37.67	452.68	40.36	453.82	43.63
Positive bias	507.24	57.08	489.45	49.43	517.26	60.23	511.04	57.10
Negative bias	495.98	78.30	513.26	81.66	483.18	71.91	490.50	78.41

*Reaction times are shown in milliseconds*.

**Table 2:** **All relevant results of the GLMs are shown here, split per latency window, and then showing the values for condition, subgroup, and the interaction between the two separately**.

	**Condition**					**Subgroup**				Condition ^*^ Subgroup					**(Significant) contrasts**					
	**Window**		***df***	***F***	**Sig**.	***N_*p*_*^2^**	***df***	***F***	**Sig**.	***N_*p*_*^2^**	**df**	***F***	**Sig**.	***N_*p*_*^2^**	**Where**	**Between**	***df***	***F***	**Sig**.	***N_*p*_*^2^**
Word	84	92	2.58	1.650	0.20	0.054	1.29	0.19	0.67	0.701	2.58	5.473	0.006	0.16	Condition ^*^ subgroup	Double vs. neutral	1.29	17.024	0.00028	0.37
	376	396	2.58	1.455	0.24	0.048	1.29	6.845	0.014	0.19	2.58	0.007	0.99	0.0004		None				
Dot	716	724	2.58	3.175	0.028	0.099	1.29	8.221	0.008	0.22	2.58	1.043	0.38	0.035	Condition	Double vs. neutral	1.29	9.006	0.005	0.237
	900	940	2.58	1.009	0.39	0.034	1.29	0.28	0.60	0.01	2.58	8.616	0.000045	0.229	Condition ^*^ subgroup	Incongruent vs. neutral	1.29	22.77	0.000048	0.44
															Condition ^*^ subgroup	Congruent vs. incongruent	1.29	20.946	0.000082	0.419

*Significant contrasts are shown as well. Note that the critical p-value is 0.05/4 = 0.0125, since four GLMs are present*.

**Figure 3:**
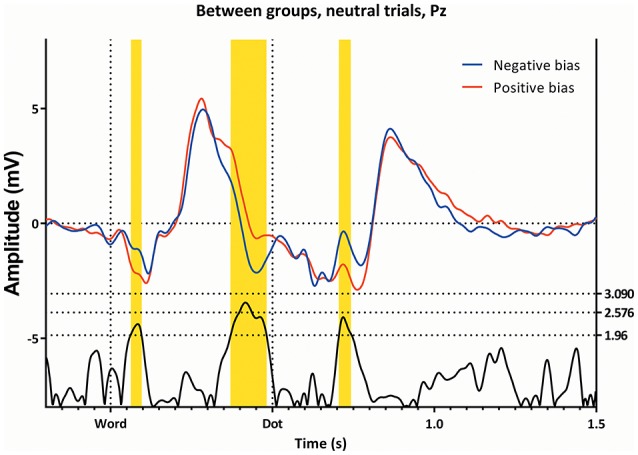
**The ERP's of the Neutral trials, as seen from Pz, for both attentional bias directions separately**. Latency window 1, 2, and 3 are marked yellow.

Figure 2 (top left) shows the mean amplitude values in the different conditions, and demonstrates the interaction between “subgroup” and “condition”; the two groups show somewhat opposing patterns, with the neutral trials showing a higher amplitude in the subgroup with a negative bias, the double trails showing a higher amplitude in the subgroup with a positive bias, while the single trails do not show a significant difference. Figure 3 shows this latency window in both subgroups for the neutral trials, where the amplitude of the subgroup with a negative bias is more negative than the amplitude of the subgroup with the positive bias.

**Figure 4:**
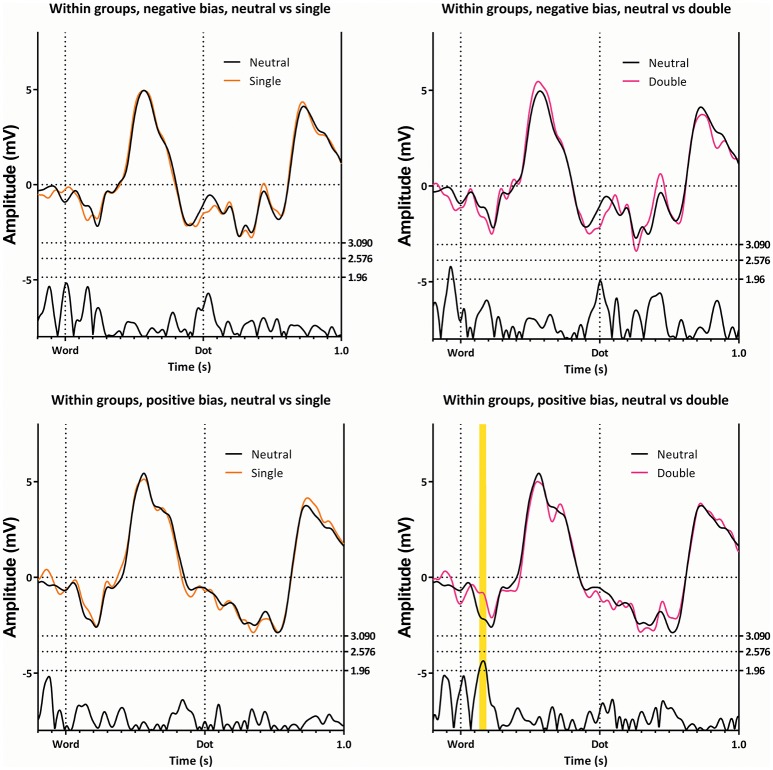
**The ERP's of the single and double trials, compared with the neutral ERP's**. The top two panes show the subgroup with the negative bias, while the bottom two panes show the subgroup with the positive bias. The comparison between the single and the neutral trials is shown on the left, while the comparison of the double and the neutral trials is shown on the right. Note the first latency window only appears on a single comparison, in a single subgroup.

Figure 4 illustrates this latency window within the subgroups. In this figure, ERPs elicited by the neutral trials are compared to the ERPs of single and double trials, in both subgroups. This figure shows differences between the double and neutral trials in the subgroup with the positive bias (lower right panel), with the double trials being less negative, but not in the subgroup showing the negative bias (upper right panel).

#### Second (late) latency window; 376–396 ms

A further comparison of the two subgroups using the t-profiles indicates the presence of one or multiple latency window(s) between 350 and 480 ms in the word-phase in all conditions. Further examination shows the window to be present in all conditions between 376 and 396 ms.

A window centering on *t* = 386 ms with a width of 20 ms was analyzed with a repeated-measures GLM, with “condition” (neutral, single, and double) within-subjects factor and “subgroup” as between-factor. As can be seen in Table 1, there is a marginal effect of “condition,” as well as an effect of “group,” yet no interaction between the two. The value of the subgroup with a negative bias is more negative than the value of the subgroup with the positive bias.

**Figure 5:**
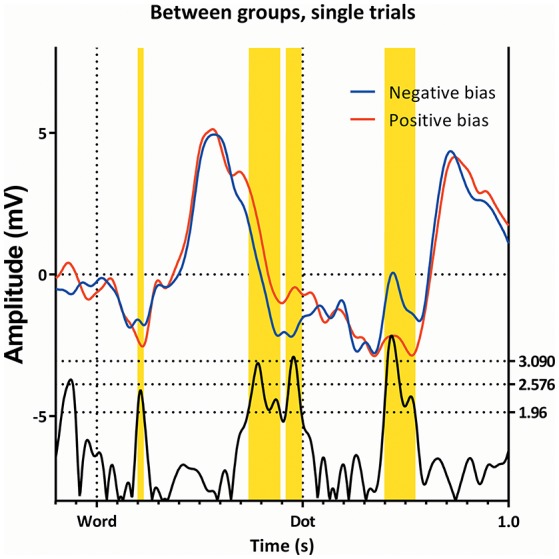
**Comparison of the single trials of the two subgroups, as ERP's**. Latency windows 1, 2, and 3 are all visible, however, note that latency window 3 is split in two by a small non-significant region.

**Figure 6:**
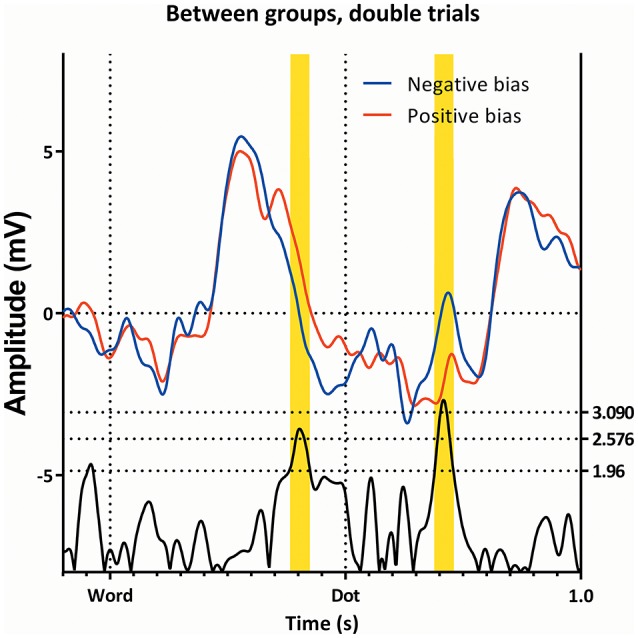
**Comparison of the single trials of the two subgroups, as ERP's**. Latency window 2 and 3 are all visible.

This latency window can be seen in Figures 3, 5, 6, where the subgroup with the positive bias shows a pronounced deflection around 350 ms, and a possibly resulting difference in slope afterwards, while the subgroup with a negative bias does not (the deflection is either small, or absent, in almost all conditions).

### Post-dot ERPs

#### Third (early) latency window: 216–224 ms

**Figure 7:**
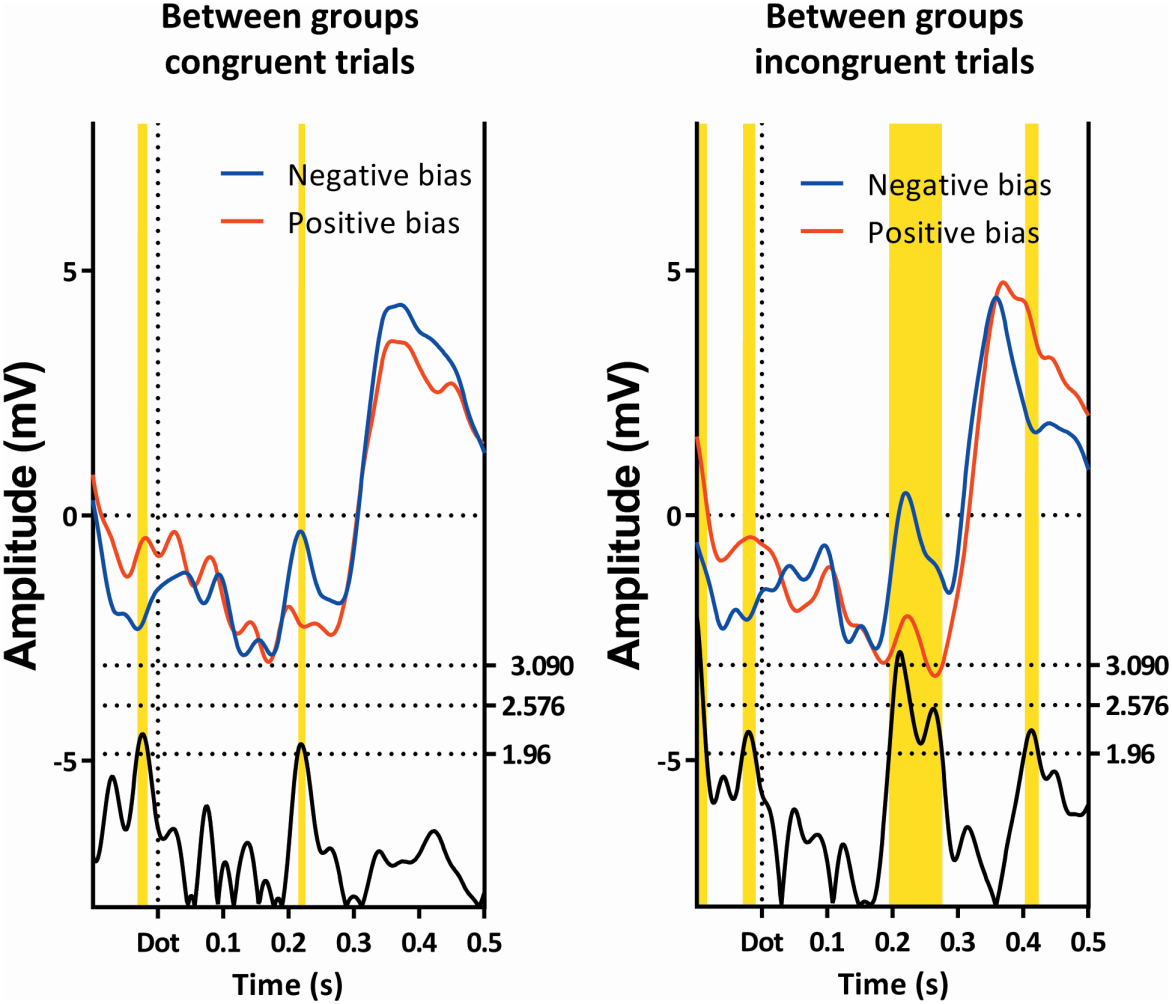
**Comparison of the congruent and incongruent trials between the two subgroups**. Latency window 3 is visible in both conditions, while latency window 4 is only visible in the incongruent trials. Note that latency window 3 is still partially visible.

At 220 ms after the appearance of the dot we find a latency window on Pz in both subgroups. This region is present in all conditions, as can be seen in Figures 3, 6, 7). This latency window is relatively narrow in the t-profiles, so a window of 10 ms is appropriate.

A repeated-measures GLM of this interval on Pz, with “condition” (neutral, congruent, incongruent, and double) as within-factor, and “subgroup” as between-factor, showed a main effect of “condition,” as well as a main effect of “subgroup,” which can be seen in Table 1. Further *post-hoc* contrasts to examine the main effect of condition suggest the most pronounced difference of “condition” to be present between the double and neutral trials.

**Figure 8:**
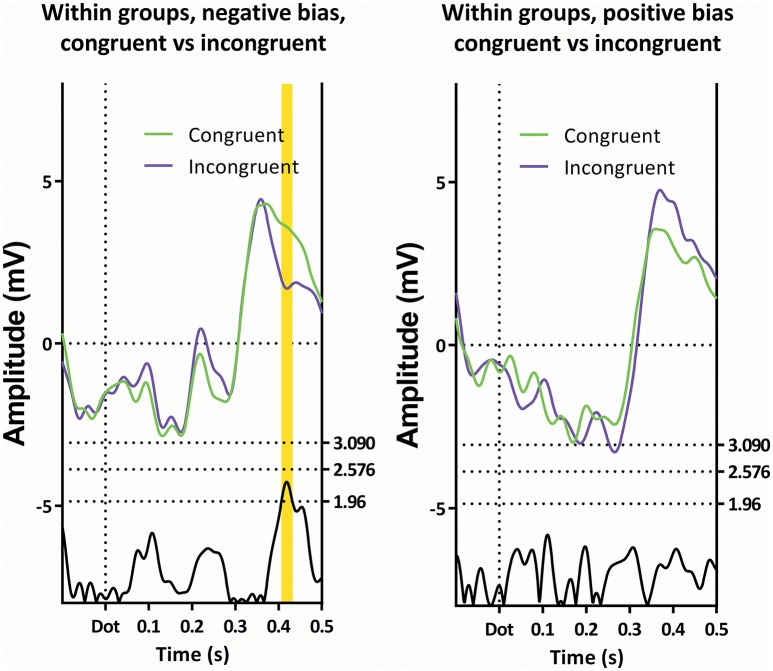
**Comparison of the congruent and incongruent trials within the two subgroups**. Only latency window 4 is visible, and then only in the subgroup showing a negative bias.

As can be seen in Figure 2, the effect of group can be explained in that the amplitude of the subgroup with a positive bias is more negative than the amplitude of the subgroup with the negative bias (this is also visible in Figures 4, 7, 8).

#### Fourth (late) latency window, 400–440 ms, negative bias

The subgroup showing a negative bias shows a region of interest on Pz 420 ms after the dot between the congruent and incongruent trials (see Figure 7).

As can be seen in Table 1, a repeated-measures GLM with “condition” (neutral, congruent, incongruent, and double) as within-subjects factor, and “subgroup” as between-subjects factor showed no effect of “condition,” nor an effect of “subgroup,” but did show an interaction between these two factors.

Within-subject contrasts suggest the strongest interaction was present in the comparison between the incongruent and neutral conditions, followed by the comparison between the congruent and incongruent conditions.

When viewing the amplitudes (see Figure 2), a pattern emerges; the differences between certain conditions seem opposite in the two subgroups. Most notably, while the subgroup with the negative bias shows the incongruent trials to have a lower amplitude than the congruent trials, the subgroup with the positive bias shows the congruent amplitude to be lower.

## Discussion and conclusion

### General

The goal of the current study was to provide support for the existence of two different attentional bias patterns for pain-related information at the neural level.

To be able to do this, we recorded the EEG during the dot-probe task. Using pain-related and neutral words as stimuli, we created congruent, incongruent, double, and neutral trials.

No differences in resting state EEG were found, suggesting that the resting states of both subgroups is similar.

### Word-phase

During the word-phase, there are three possible conditions, based on the properties of the words involved; neutral, single, or double. As the dot has not appeared at this point, there is no congruency information, meaning the only manipulation here is the level of saliency, or relevance, of the trial.

Very quickly after the words appeared (around 88 ms, first latency window), the two subgroups diverge, with the subgroup showing the positive attentional bias (signifying increased vigilance) having a more negative deflection than the subgroup showing the negative attentional bias (signifying avoidance), in the neutral and single trials.

The deflection in this window falls within the classic P1-N1 domain. The amplitude of this component has been known to vary if the participant is instructed to direct attention toward the stimuli (Haider et al., 1964; Naatanen, 1975), and increased top-down attention increases the amplitude of this component (Legrain et al., 2002; Lee et al., 2009; Van der Lubbe et al., 2012). It has been suggested to be reflective of a frontal sensory gain control mechanism (Luck et al., 2000), which would make it reflective of a top-down control mechanism with the goal of priming the participant. The frontal lobe has been implicated in top-down somatosensory priming before, which is reflected by relatively early components (Wang et al., 2014), such as the components involved in the P1-N1 domain.

As the subgroup showing a positive attentional bias can be seen as having “increased vigilance,” this makes sense; increased vigilance can be read as the subject “priming” itself using frontal control systems, which it does by pre-allocating more attentional resources to the processing of the words, leading to an increased N1.

This does not explain why this difference also appears on the neutral trials, which shouldn't have any relevance due to their low saliency. Assuming the neutral trials have negligible saliency (this potential issue will be addressed under “limitations”), one explanation may be that the subgroup with a positive attentional bias might display a heightened state of arousal, or attribute additional relevance to words in general, while the other subgroup is not, or might even be predisposed to direction attention from the complete task (i.e., avoiding the task, by lowering the relevance of the words).

The fact that this group difference was most pronounced for the neutral trials, can be explained by differences in the number of trials; the neutral trials number in the hundreds, while each participant is exposed to 30 double trials. The “single” trials are made up of all trials with a single pain-related word; they are made up of 30 congruent and 30 incongruent trials, as the distinction between the two is non-existent before the dot appears. As a result, the level of noise differs per trial type, with the neutral trials being very low, and the double trials being the highest in noise.

The two groups diverge again on the P3-N4 window (between 350 and 480 ms, second latency window) with the subgroup showing the positive attentional bias having an overall higher amplitude when compared with the subgroup showing the negative attentional bias.

There appear to be two deflections within this latency window; one around 386 ms (which can be interpreted as a P3b), and one around 478 ms (which can be interpreted as a N450).

However, upon visual examination of the ERPs it seems likely that the perturbation introduced by the first deflection extends into the region of the second deflection. Moreover, the first deflection (the presumed P3b) seems absent in the subgroup showing a negative attentional bias on visual inspection.

Regardless, the two subgroups differ on the P3b, with the subgroup showing a positive attentional bias having a more pronounced P3b in every condition. As the P3b has been associated with event categorization, attention and memory processing, and target evaluation (Kok, 2001; Polich, 2003, 2007), and the subgroup in which the P3b is more prominent is the subgroup showing the positive attentional bias (which is also known as “hypervigilant”), this would suggest that this subgroup allocates more (attentional) resources to the processing of the stimuli. Similar results have been found before (Bar-Haim et al., 2005). This might be related to the earlier mentioned frontal control systems; not only are the somatosensory components enlarged by priming, but also the later evaluation processing. Moreover, this is consistent with earlier findings (Wang et al., 2014).

### Post-dot

After the disappearance of the words, the participant is to respond to the location of the newly-appeared dot. The preceding conditions (neutral, single, double), combined with the dot location, creates four trial types; neutral, congruent (single pain-related word, with the dot on the pain-related word), incongruent (single pain-related word, with the dot on the neutral word), and double trials.

Around 220 ms after appearance of the dot, the two groups diverge on all conditions. In this window (around 220 ms, third latency window), a peak appears, with the subgroup showing the negative attentional bias having a more positive peak. This interval is where the P200 is expected.

This has been found before, in anxious vs. non-anxious individuals (Eldar et al., 2010), which was explained as an increased commitment of attentional resources. As the P200 is more or less established as reflecting selective attention and item encoding, and is commonly associated with frontal top-down control (Dunn et al., 1998), this is a plausible explanation for the differences between subgroups. The subgroup with a positive attentional bias is marked by being drawn toward the location of the screen that showed pain-related words, while the other subgroup is trying to pull away from this location, which might require additional attentional resources. It is interesting to note that this is also true for the neutral words, which do not incorporate pain-related words.

Finally, there were differences in the subgroup showing a negative attentional bias around 420 ms after appearance of the dot. This is a region commonly associated with the P3b. As this deflection is maximal around the parietal region, which is consistent with literature for the P3b (Polich, 2007), it is likely to be indeed the P3b.

In this latency window, the subgroup displaying a negative attentional bias showed a more pronounced P3b in the incongruent trials when compared with congruent trials, while the subgroup with the positive attentional bias seems to show the opposite effect. This is especially apparent when observing Figure 2, where the ERP amplitudes follow a specific pattern, which is reminiscent of the RT data on the dot-probe task. The subgroup showing a negative attentional bias is characterized by avoidance-like behavior, which means they react slower on congruent trials, and faster on incongruent trials. The subgroup showing a positive attentional bias is characterized by increased vigilance which means they react faster on congruent trials and/or slower on incongruent trials.

### Limitations

Although, the current study clearly shows differences in processing between the two subgroups using EEG, some limitations need to be discussed.

While the ERP's of the congruent, incongruent, and double trials are still quite sufficient, the difference in quality is obvious when viewing the grand averages of the neutral trials; these are the result of large amounts of trials, and are practically noise-free. While this discrepancy is not expected to have any consequences for our conclusions, it should still be said that future experiments would benefit from larger trial numbers in the non-neutral conditions.

The employed method does have a drawback, in that potential interesting differences are missed, which is illustrated by investigating Figure 8. Here, the subgroup showing a positive attentional bias does not show significance on the t-profiles between the congruent and incongruent trials. It does show an interesting difference around 770 ms (270 ms after the dot) on Pz, where a peak has a visibly larger amplitude in the congruent condition when compared with the incongruent condition. This is especially striking, since the preceding negative peak shows the opposite effect, where the amplitude is reduced in the congruent condition. Moreover, this peak is recognizable as a P3a, which has been associated with frontal stimulus-driven attentional systems (Polich, 2007).

Classical methods would ignore these t-profile-based latency windows, and simply utilize literature-based intervals, or manually pick peaks while possibly calculating difference scores between this peak and the preceding negative peak. For example; the positive deflection just before latency window 4 could be combined with its preceding negative deflection in a peak-to-peak-method, which could yield statistically significant results. The benefit of the currently employed approach is that it is highly robust, however, it is insensitive to this specific presentation, and therefore these deflections may be unjustly ignored.

Another potential limitation concerns the reliability of the dot-probe paradigm (Kappenman et al., 2014). One of the few studies on this particular subject yielded only very little significant results (Dittmar et al., 2011), suggesting either the dot-probe paradigm is flawed, or the effects of attentional biases are spurious findings. However, given the wealth of studies utilizing the dot-probe paradigm to good effect (e.g., demonstrating that specific attentional biases predict future conditions, such as postoperative pain), as well as the studies showing attentional biases in other populations, and the success of this study, we would argue that the dot-probe paradigm as well as attentional biases can be made visible in a reliable manner.

The dot-probe paradigm, while used often, can introduce a limitation; participants have been known to simply not read the words, or to have erroneous (from the perspective of the study) associations with the used words. In the current study, we tried to minimize these potential limiting factors by including a questionnaire testing their memory and perception of the words, which was applied after completion of the test session. This test showed the participants did indeed read and remember the words.

Still, the words might not activate the pain schemata associated with pain (Crombez et al., 2013), even though the subject processes the words properly. The fact that there are significant differences between different types of trials (congruent vs. neutral, for example) suggests that this is not the case, as subjects process the words sufficiently to evoke these differences, but this does not exclude the possibility that the activation is different from real-world examples of pain.

Additionally, the choice of words can impose limitations as well. In this study, we chose to implement pain-related words, but these might not seamlessly overlap with aversive stimuli. However, we feel this is an appropriate choice as pain-related information is highly aversive. While a broader set of words might cover all possible aspects of aversive information, it would also include additional noise and increase the duration of the experiment.

### Future research

Attention is frequently studied using imaging techniques, and as a result there is much known about which areas of the brain contribute to the phenomena of attention (Knudsen, 2007). The separate deflections of EEG can relate to specific brain areas, such as the frontal eye fields, which are areas involved in mediating task-specific functions, and the posterior intraparietal sulcus, which varies its activity with the level or intensity of attention involved (Culham et al., 2001). Other regions, such as the thalamus, dorsolateral prefrontal cortex, and the basal forebrain are also part of the attentional networks, and fulfill distinct, yet partially unknown, roles (Small et al., 2005). Knowing which regions are active at those deflections can assist in interpretation of these deflections and their underlying phenomena.

However, due to the limited number of EEG channels in the current study, it is not possible to relate ERP activity to any of brain areas, using just the data gathered in this study. As such, we would suggest to include fMRI experiments, or to expand the number of EEG-channels, to allow for source localization in a future study.

### In conclusion

In this study, we demonstrated that different attentional biases exist in the healthy population, by showing differences in ERP's. Most notably, the deflections associated with early and late attentional components, including the P3B, as well as a positive deflection in the timeframe of proposed response evaluation processes differ significantly between subgroups.

Moreover, these two biases do not only differ on trials utilizing pain-related words, but also on neutral trials, which suggests there are fundamental differences between these groups in processing words in general. Previously, it has been shown that these two attentional biases can be associated with different response patterns on questionnaires, but now we show that they also differ in basic neural phenomena.

Most interestingly, while the participants are split based on the bias index, which is calculated based on their response times on the dot, we already see significant differences between the groups before the dot appears (i.e., during the word processing phase). This suggests that the two attentional bias groups represent genuine differences in the processing of words, which can already be detected at the word-processing level.

This information is of crucial importance as these biases have been associated with, among other things, the risk of future pain chronification (Lautenbacher et al., 2010). Further investigation into these attentional biases and their effects is expected to yield not just more information regarding the effects of these biases, but also possibly handles for future treatment, as well as a deeper understanding of the underlying phenomena.

## Ethics statement

This study was approved by the Ethic Committee Social Sciences of the Radboud University Nijmegen, in accordance with the requirements of the Declaration of Helsinki. The approval is registered under ECG2012-1301-005. All subjects signed a standard written informed consent, as used by the Donders Institute for Brain, Cognition and Behavior and the Faculty of Social Sciences of the Radboud University Nijmegen.

## Author contributions

The basic study design was made by CMvR, JO, and CHvH. The practical work was primarily done by CHvH and KdK. EEG analysis and interpretation was done by CMvR, MJ, KdK, and CHvH. All authors contributed equally to the writing of the article.

### Conflict of interest statement

The authors declare that the research was conducted in the absence of any commercial or financial relationships that could be construed as a potential conflict of interest.
