# Supplementary material for: Evidence for a Priori Existence of Attentional Bias Subgroups in Emotional Processing of Aversive Stimuli
Source: Front Behav Neurosci. 2017 May 12;11:87. doi: 10.3389/fnbeh.2017.00087 (PMC5427543; doi:10.3389/fnbeh.2017.00087)
Supplement: Supplementary file 1 [file Table1.docx]

# Pain (1)

- kloppend
- bonzend
- barstend
- hinderlijk
- flitsend
- schietend
- prikkend
- stekend
- doorborend
- snijdend
- messcherp
- drukkend
- knellend
- snoerend
- trekkend
- splijtend
- scheurend
- branderig
- brandend
- broeiend
- gloeiend
- vreselijk
- vervelend
- tintelend
- ellendig
- elektrisch
- krampend
- zeurend
- knagend
- ontzettend

# Pain (2)

- negatief
- ongelukkig
- verachtend
- verloren
- zenuwachtig
- geweldadig
- geirriteerd
- schuldig
- bedroevend
- verdrietig
- nadelig
- treurig
- chagrijnig
- hatelijk
- onvriendelijk
- hopeloos
- rampzalig
- kattig
- gespannen
- verachtend
- minachtend
- waardeloos
- onbetrouwbaar
- prikkelbaar
- onbeleefd
- vijandig
- intolerant
- destructief
- arrogant
- chaotisch

# Neutral

- aandachtig
- aannemelijk
- aanvaardbaar
- aanzienlijk
- abstract
- acceptabel
- aftrekbaar
- afzonderlijk
- alledaagse
- auditief
- authentiek
- authentiek
- begrijpelijk
- behoedzaam
- bekwaam
- benodigde
- beschikbaar
- bevoegd
- bewolkt
- binnenlands
- biologisch
- bovengronds
- bruikbaar
- competent
- concreet
- consequent
- consistent
- conventioneel
- cultureel
- denkbaar
- diagonaal
- diepzinnig
- doordeweeks
- draagbaar
- dynamisch
- economisch
- eenmalig
- eigenaardig
- eigenlijk
- elektronisch
- exclusief
- experimenteel
- expliciet
- explosief
- flexibel
- fotografisch
- functioneel
- fundamenteel
- fysisch
- gangbaar
- gebogen
- gebruikelijk
- gekromd
- geleidelijk
- gelijkmatig
- gematigd
- gemiddeld
- gerimpeld
- gevouwen
- grenzeloos
- handmatig
- herhaaldelijk
- herkenbaar
- hermetisch
- historisch
- hoekig
- horizontaal
- huidig
- huishoudelijk
- imposant
- indirect
- industrieel
- informatief
- informeel
- innerlijk
- katoenen
- klassiek
- knapperig
- koloniaal
- kolossaal
- kringvormig
- kunstmatig
- landelijk
- leidend
- levensecht
- lichtelijk
- liggend
- lopend
- luisterend
- magnetisch
- manueel
- massief
- mechanisch
- methodisch
- middelbaar
- middelste
- moleculair
- muzikaal
- nadrukkelijk
- nauwgezet
- nauwkeurig
- nauwlettend
- neutraal
- nietszeggend
- nostalgisch
- objectief
- obscuur
- omliggende
- onbeperkt
- onbruikbaar
- ondergronds
- onderling
- onderste
- ondeugend
- ongekend
- ongezien
- onherkenbaar
- onopvallend
- onpartijdig
- onregelmatig
- onveranderd
- onzijdig
- openbaar
- opmerkelijk
- organisch
- overwegend
- parallel
- particulier
- passend
- permanent
- plaatselijk
- plastisch
- primaire
- principieel
- productief
- punctueel
- razendsnel
- recent
- recentelijk
- rechtshandig
- regelmatig
- regionaal
- relatief
- resoluut
- reusachtig
- rijdend
- roestvrij
- schijnbaar
- schriftelijk
- sluitend
- soortgelijk
- specifiek
- sportief
- spraakzaam
- sprekend
- startklaar
- symbolisch
- symmetrisch
- synthetisch
- systematisch
- talrijke
- tamelijk
- tastbaar
- terzake
- theoretisch
- toonbaar
- traceerbaar
- transparant
- treffend
- typisch
- uitdrukkelijk
- uitgebreid
- uitvoerig
- universeel
- veelvuldig
- verbaal
- verkrijgbaar
- versteende
- verticaal
- verzameld
- verzekerd
- visueel
- vloeibaar
- volstrekt
- voorspelbaar
- voorspellend
- vredig
- vreedzaam
- wekelijks
- wereldwijd
- wezenlijk
- wiskundig
- zakelijk
- zekerwetend
- zinloos
- zwemmend
- zwijgzaam
- gedetailleerd
- gelijktijdig
- globaal
- hoofdzakelijk
- inbegrepen
- middeleeuws
- naamloos
- vergezocht
- vermeend
- vluchtig
- volkoren
- zeewaardig
